# Assessing the Efficacy of Mobile Health Apps Using the Basic Principles of Cognitive Behavioral Therapy: Systematic Review

**DOI:** 10.2196/jmir.8598

**Published:** 2017-11-28

**Authors:** Amy Leigh Rathbone, Laura Clarry, Julie Prescott

**Affiliations:** ^1^ Department of Education and Psychology University of Bolton Bolton United Kingdom

**Keywords:** cognitive therapy, behavior, mHealth, mobile, phone, health, treatment efficacy, intervention study, randomized controlled trial, review, systematic

## Abstract

**Background:**

Cognitive behavioral therapy (CBT) in its basic principle has developed itself as a stand-alone, substantial method of therapy. With effective application in therapy for a range of mental health issues, the spread of CBT methods to Web-based therapy sources is evident. The development of mobile phone apps using CBT principles is increasing within the research area. Despite the move to Web-based methods of therapy, it is argued that these methods lack the same efficacy of face-to-face therapy sessions.

**Objective:**

The aim of this review was to assess extent research findings with regard to the effectiveness

of CBT-related mobile health (mHealth) apps. By assessing only studies employing a randomized controlled

trial design, the review aimed to determine app efficacy within the highly regarded method of

investigation.

**Methods:**

A comprehensive literature search was conducted across several databases. Search results were

filtered, and results were subject to strict inclusion and exclusion criteria because of the nature of the

review. Where possible, analysis of effect size was calculated and results reported.

**Results:**

A total of 8 studies investigating the effectiveness of mHealth CBT-related apps across a range of

mental health issues were reviewed. Three studies used the app against a control group, and 5 studies

used the app intervention against another form of treatment or intervention. A range of effect sizes were

seen across all included studies (*d*=−0.13 to 1.83; 0.03-1.44), with the largest effects often being seen when comparing the data from pre- to posttest for the app engaged group.

**Conclusions:**

The studies reviewed support the use of mHealth apps containing CBT principles for a range

of mental health issues. However, the effectiveness over longer time periods should be

assessed. Researchers and professionals should seek to collaborate effectively when creating new apps

to enhance their effectiveness as a treatment for the general public.

## Introduction

### Cognitive Behavioral Therapy

Cognitive behavioral therapy (CBT) is a substantially adapted, personalized, psychosocial therapy [1]. It has emerged as a viable, empirically reinforced treatment for various mental health issues [2]. The therapy proposes maladaptive cognition correlates to a cognitive and attention bias toward misinterpretation of information and perceiving certain information to be directly threatening and cataclysmic [3]. CBT focuses on personal connotations added to situations and produces empowering psychological strategies to reevaluate the meanings attributed to situations, promoting learned practice for more positively altered behaviors, emotions, and thoughts [4].

Beck et al [5] theorized that, “Cognitions (verbal or pictorial ‘events’ in a person’s stream of consciousness) are based on attitudes or assumptions (schemas), developed from previous experiences.”

Beck et al [6] went on to add, “The psychological sequence progresses from evaluation to affective and motivational arousal, and finally to selection, and implementation of a relevant strategy.”

These two short quotes encompass the basic principles of CBT. CBT consists of three core principles: cognitive activity affects behavior, cognitive activity may be monitored and altered, and desired behavioral change can occur through cognitive change [7]. The therapy aims to target negative emotions that can not only be overwhelming but can also have a detrimental effect on a person’s quality of life. Negative emotions, when experienced in the correct context, can be a typical occurrence, for example, stress, bereavement, anger, or jealousy. However, if these feelings increase and occur exponentially, physical symptoms such as increased blood pressure, headaches, insomnia, and loss of libido can develop.

The therapy itself is tailored to meet the patients’ needs and aims to utilize the previously formed therapeutic alliance between patient and therapist to recognize and comprehend present difficulties [8]. Patients are required to engage with *homework* outside of therapeutic hours. This encourages patients to foster a sense of cognitive self-awareness away from a clinical setting.

### Applications and Efficacy

CBT can be applied to a variety of mental health issues and when utilized in the proper manner, or comorbid with other relevant treatment, result in a significant reduction in symptomology. One such issue is obsessive compulsive disorder (OCD).

Recent research has found that CBT has the potential to reduce and control symptomology of OCD in a way that far exceeds the pharmacological methods such as serotonergic antidepressants [9]. Several studies found that CBT garnered a greater effect size and provided more substantial improvements of clinical symptoms [10-13]. A meta-analysis carried out by Olatunji et al [14] examined the efficacy of CBT for OCD and found that the therapy was highly effective for the reduction of symptomology. The study found that there were large effect sizes immediately post treatment and medium effect sizes during a follow-up.

CBT has shown effectiveness for treating both children [15] and adults [16] suffering from posttraumatic stress disorder (PTSD). CBT specifically used for PTSD tends to be trauma-focused. Trauma-focused CBT (TF-CBT) is a highly versatile model of psychotherapy that focuses on trauma-specific emotional stimulus [17]. When implementing CBT for PTSD in a randomized controlled trial (RCT), Smith et al [18] found that after a month-long symptom observation period, 24% of young people with a preliminary diagnosis of PTSD developed so much that they actually failed to meet the criterion for the disorder. The same study also found that TF-CBT reduced symptoms of not only PTSD but depression and anxiety and also enabled a better quality of life. These results were also maintained over a span of 6 months.

Previous studies and meta-analyses have shown that CBT is an efficacious therapy for anxiety disorders. There have been results showing that CBT has the benefit of reducing comorbidity of multiple anxiety disorders [19]; its clinical utility is valid when treating anxious children and avoiding relapse in adolescents and adulthood [20] and overall reduction of anxiety disorder symptomology [21].

CBT has shown positive results in many other areas of mental health, reinforcing the therapy’s applicability. For example, CBT has been used to assist people with irrational phobias to alter their judgment toward and aversion of fear-instilling stimuli [22], as an antidepressive treatment [23], to treat eating disorders [24,25], and many more.

### Accessing CBT

More typically, CBT has been delivered in a clinical environment by a therapist who meets face-to-face with a patient. However, this method can present patients with additional obstacles for various reasons. This could be because of time constraints, prior engagements, or misguided prioritization of well-being. Some patients may suffer with social anxiety, agoraphobia, or physical issues that inhibit them from leaving their home.

There are emergent deliverance methods of CBT being explored, such as telephone CBT [26,27], CBT delivered via texting [28], and Internet-based CBT (iCBT). With the introduction of iCBT, patients became able to communicate and confer with their therapist via a Web-based platform, and treatment can be delivered using Web-based programs.

Although it has been argued that iCBT lacks in efficacy because of the absence of face-to-face interaction with a therapist, Carlbring et al [29] carried out a study that evidenced support for Web-based distribution of treatment for panic disorder because of the subsequent large effect sizes. In this study, face-to-face meetings were substituted with short, once weekly phone calls. The sample consisted of 60 participants who met the criteria for panic disorder as categorized by the *Diagnostic and Statistical Manual of Mental Disorders, 4th edition*. The study measured aspects such as cognitions, physiological sensations experienced by patients with anxiety disorders, degree of agoraphobia, generalized anxiety, level of depression, and quality of life. The study spanned over 10 weeks when follow-up data was collected. Follow-up data was also collected at 9 months post study. Results show that the intervention group improved significantly in all measures between pre- and posttreatment (*t*_29_=4.4-8.7, all *P*<.001). Another study asked participants their opinions of iCBT. Of 105 participants, 76.2% (80/105) reported that they had not particularly felt the absence of face-to-face contact with a therapist, and 94.3% (99/105) would recommend the method to others [30].

Access to iCBT equips patients with the provisos and skills to guide their own therapy. Haarhoff and Kazantzis [31] contested that several components of CBT, such as observing and altering one’s own beliefs, feelings, and conduct, can be considered as salient self-help techniques. When completing *homework* that has been set by a CBT therapist, a patient is essentially contributing to their own well-being using a self-help nature.

Naeem et al [32] contests that self-guided CBT (SG-CBT) can be used as an intervention with minimum direct contact. For patients in need of CBT who face obstacles hindering face-to-face attendance, a self-guided platform to access therapy holds many advantages. Previous studies have shown that SG-CBT can instigate and maintain significant clinical improvements [33]. SG-CBT can enable the avoidance of using mental health services, divert from long waiting lists, bypass obstacles that prevent the use of service, and effectively lower the costs of care for both patients and amenities.

The ubiquity of mobile phones today has become the norm for society. Simultaneously, it is estimated that over 8 billion individuals are connected to the World Wide Web using mobile phones as a platform [34]. Due to the copiousness of mobile phones, the utility of mobile health (mHealth) has never been more endemic. Studies have found that 31% of mobile phone owners use them to access health information; 19% have also installed a mobile app that relates to current medical condition or to manage their health and well-being [35,36].

Some mental health and well-being–focused apps are now integrating mobile technology with CBT by using the basic principles and underpinnings of CBT to enhance the outcome of self-guided therapy. As CBT is an umbrella term for various cognitive and behavioral therapies, the therapeutic content of said apps vary.

### Aim

Both the increasing use and pervasiveness of mobile phones and the integration of basic CBT principles into mHealth apps are cause for concern because of the clinically unsupervised nature of the intervention and subsequent outcomes. This review aims to synthesize the extant literature and assess the efficacy and user experience of SG-CBT apps with reference to app content. This review followed the guidelines provided by preferred reporting items for systematic reviews and meta-analyses (PRISMA) and adhered to the published PRISMA checklist [37].

## Methods

### Search Strategy and Selection of Studies

A comprehensive literature search in relevant bibliographic, Web-based databases was carried out (PsycARTICLES, Google Scholar, Scopus, ProQuest Central, JMIR, and PubMed). Initially, search terms used were not restricted to the title only. They were found within the title, abstract, or full paper. Words searched were “cognitive,” “behaviour,” “behavioural,” “therapy,” “CBT,” “mobile phone(s),” “application,” “randomised,” “controlled,” “trial,” and “RCT.” The conjunction “AND” and the logical operator “OR” were also used in the search terms (Multimedia Appendix 1). The searches were consistently inclusive of “randomised,” “controlled,” “trial” and “RCT.” This ensured that the search did not return studies of other experimental designs. These words were searched because of their direct link with the topical therapy and chosen study design under review.

A vast amount of the search terms were used because of their inclusion in the medical subject headings database. The search terms for this systematic review were stringent because of the focus on one method of therapy, one method of delivery, and one method of experimental design. The reason for this is that the current cultural climate dictates less and less time for individuals to enhance their well-being in a physical setting; therefore, the reliance upon mHealth apps and their instant gratification increases. Moreover, the readily available apps are more beneficial to those suffering from debilitating social phobias or physical ailments. RCTs were the only experimental design reviewed because of the fact that results of the study are reported in a way which sees as many biases removed as possible [38].

From the papers returned by the initial search, abstracts were read to check eligibility. If the abstract of the paper was deemed irrelevant, the paper was retracted from further analyses. If the abstracts were relevant, full text of the paper was then reviewed for eligibility. Papers were discarded if they met the exclusion criteria. Eligible, full text papers had the reference section screened to find further relevant papers. Unpublished studies, dissertations, and gray literature were neither sourced nor included in this review.

The second author carried out the literature search and elected the inclusive papers. The first author and the third author then considered and agreed with the final included studies. The first and the second author carried out the risk of bias assessment.

### Inclusion Criteria

The inclusion criterion for the systematic review was inflexible. The studies had to be reported in English, and they ranged from January 2008 to May 2017. This is owing to the fact that apps were only released in 2008 [39]. All studies were required to contain an app that used the basic principles of CBT with an RCT design. As highlighted within the search strategy section, studies reviewed were restricted to those of an RCT design based on their data reporting methods and the comprehensive removal of bias. All studies were reported in peer-reviewed, scholarly journal articles. There were no demographic restrictions. The field of study was restricted to that of mHealth.

### Exclusion Criteria

Studies devoid of actual apps were excluded as they were irrelevant to the focus of the review, for example, proposals, reviews, meta-analyses, conferences, and case studies. Dissertations, secondary sources, and gray literature also met the exclusion criteria. This was to ensure that all studies included had an international standard RCT number, essentially meeting the criteria of Consolidated Standards of Reporting Trials [38].

### Primary Outcome Measures

This review looked at apps that aim to deliver therapy using the basic principles of CBT and encourage self-guided improvement of one’s well-being. The primary outcome measures were the initial efficacy of the intervention and the overall user experience of the app itself.

### Analyses of Effect Sizes

Where the data was attainable, between-group and within-group effect sizes (Cohen *d*) were established using the variance between the pretest and posttest results (within-group effect size) or the variance between the control and intervention group posttest results (between-group effect size) and dividing by the pooled standard deviation. Effect sizes of 0.2 are deemed relatively small. Effect sizes of 0.5 are deemed to be moderate, and those of 0.8 or higher can assume to be associated with large effect sizes [40,41].

### Quality Assessment

The overall qualities of included studies were assessed using the Cochrane risk of bias assessment tool [42]. The tool checks studies for biases such as random sequence generation, allocation concealment, incomplete outcome data, selective reporting, and other biases (Multimedia Appendix 2). The blinding criteria of the Cochrane risk of bias assessment tool were not studied as the criteria are almost impossible to abide by when implementing mental health interventions [43]. The Cochrane risk of bias assessment has been highlighted as the most appropriate method of quality assessment for the RCT design studies [44].

## Results

### Included Papers

**Figure 1:**
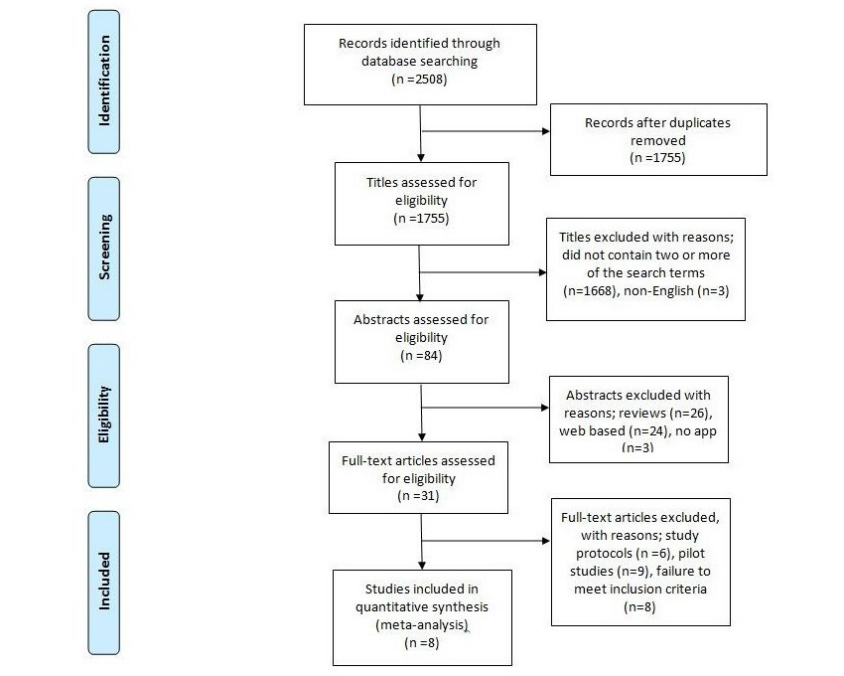
Preferred reporting items for systematic reviews and meta-analyses (PRISMA) flow diagram.

An amassed total of 2508 records were considered through search identification. After removing duplicate records, a total of 1755 records were considered by their title alone. Records were excluded if their title did not contain one or more of the utilized search terms (n=1668). Non-English papers were also excluded at this stage (n=3). Abstracts were then assessed for eligibility, with papers being excluded if they were reviews, Web-based studies, or studies that contained no information of an app being used. The remaining articles (n=31) were deemed eligible for full text review. Following the full text reviews, a further 23 papers were removed because of being pilot studies, study protocols, or failing to meet inclusion criteria. This left a total of 8 studies eligible for review as presented in Figure 1.

### Study and Intervention Characteristics

Across all studies there were an overall total of 1794 participants. Each of the included studies utilized an app, 3 of which were pitted against a control group. The remaining 5 studies used the app intervention against another form of treatment or intervention. Some mHealth interventions have remained unnamed within the studies (n=4), whereas others have been coined (n=4).

All studies (n=8) measured psychological outcomes such as depression (n=4), chronic pain acceptance (n=1), insomnia severity (n=1), stress (n=1), and PTSD symptoms (n=1).  Multimedia Appendix 3 provides a comprehensive overview of all inclusive study characteristics.

### Risk of Bias

All of the studies employed an RCT design and were overall, deemed to be of low to moderate risk when assessed against Cochrane risk of bias assessment tool. However, there were some studies that had elements of risk.

Some studies failed to report their study’s random sequence generation (n=4). Due to this, it was unclear as to whether the appropriate procedure had been adhered to [45-48].

The Cochrane’s risk of bias assessment helped researchers to identify further biases. For example, in 2 studies, participants were offered monetary incentives, potentially altering the demand characteristics [47,49]. Another study contained an unequal gender split [45].

The majority of the studies were low risk for the outcome data assessment; however, Whittaker et al’s [48] study was deemed to be potentially high risk as only a relatively small fraction of participants read the interventional messages in their entirety. Horsch et al’s [50] study had a high attrition rate, and follow-up data collection was taken for the intervention group but not collected for the wait-list control group. Therefore, no comparison could be made for the longer term effects of the intervention. Overall, the studies had high response rates and strong engagement, evidenced in Multimedia Appendix 3.

In this review, all studies employed an RCT design to measure the efficacy of CBT mHealth interventions. Across inclusive studies, the range of the within-group and between-group effect sizes were −0.13 to 1.83 at posttest and 0.03 to 1.44 at follow-up. The largest effects were often seen when comparing the data from pretest to posttest for the app engaged group. These effect sizes diminished at follow-up data collection dependent upon length between posttest and follow-up.

### Efficacy

The inclusive studies provide promising results with regard to the integration of the basic principles of CBT in mobile phone apps for mental health. The Sleepcare app, devised by Horsch et al [50], was the first of its kind. The app used CBT for insomnia (CBT-I) via a diary method that aimed to treat the symptoms of insomnia severity. This was to increase access to treatment. A significant interaction of the app was found (*P*<.01), and there was a large effect on insomnia therapy (*d*=−0.66). A significant effect upon insomnia severity was also recorded at a 3-month follow-up.

One study carried out by Kuhn et al [49] aimed to meet the needs of the population who suffered with PTSD but were not receiving treatment, by offering them psychoeducational information and evidence-based cognitive behavioral coping tools through an app. Post treatment, participants who used the app as an intervention had significant improvements in PTSD symptoms (*P*=.04), depression symptoms (*P*=.005), and psychosocial functioning (*P*=.007). However, at follow-up tests, it was found that there was no difference between the wait-list participants and the intervention participants. This shows that although the initial results were significant, there was no longevity of efficacy.

Kristjánsdóttir et al [51] found a dearth in the research of chronic pain acceptance and mobile phone self-guided support. Their study explored the efficacy of their unnamed app that was used for 4 weeks and consisted of a diary format and therapist feedback following a chronic pain rehabilitation program. The intervention significantly improved pain acceptance, lowered the catastrophizing of pain, and had a large effect size (*d*=0.87). At 5 months posttest, the between-group effect sizes were still moderate (*d*=0.74, *P*=.003), acceptance of pain (*d*=0.54, *P*=.02), and functioning and symptom levels (*d*=0.75, *P*=.001). However, it cannot be specified whether the effect sizes were because of the app itself, therapist feedback, or even the rehabilitation program. The effects could be because of the combined participation in each method of therapy.

Ly et al [52] used an unnamed app that produced moderate within-group effects on stress (Perceived Stress Scale [PSS]: *d*=0.50) and a moderate to large effect between groups (PSS: *d*=0.62).

Of the 8 studies, half aimed to treat the symptoms of depression (n=4). In a noninferiority trial, Ly et al [45] compared blended treatment, which consisted of a mobile phone and face-to-face therapy, against full behavioral activation treatment for depression. Although the study gave no conclusive findings with regard to the more efficacious treatment, it did find that the blended approach could treat up to twice as many patients because of the ease of access to the app. The MoodHacker app [47] had a significant effect on depression and negative thoughts, and the app MEMO [48] had a large, significant between-group effect on increased positivity when using CBT.

Overall, the collated studies evidenced that CBT delivered via the medium of mobile phones apps, utilized in a self-guided manner, can be efficacious. Half of the studies aimed to treat depression, with generally positive results. mHealth CBT aided the reduction of scaled depressive symptoms and negative thoughts. However, it is salient to note that these results did not maintain significance unless there was continuity of engagement with the app. It was also found that using standard CBT in conjunction with mobile phone deliverance enabled a wider dispersal of the therapy. This is because of the fact that mobile phones are easier to access than health care settings. Both apps that investigated the treatment of PTSD and chronic pain acceptance were innovative and focused upon coping strategies. This suggests that a participant’s engagement with the therapy, willingness, and ability to access mHealth platforms is key to the efficacy of CBT delivered using this method.

### User Experience

Of the 36 participants that Ly et al [52] included in their study, only 16 adhered to the intervention for the full time span of 6 weeks. In Birney et al’s [47] study, participants who received the MoodHacker app intervention used it an average of 16 times (standard deviation [SD] 13.3), which totaled an average of 1.3 hours usage across the study (SD 1.3). A total of 82.4% found the MEMO app [48] to be useful and stated they would reuse it. A significant proportion of the intervention sample (90.7%) said that they would recommend the app to a friend. The Sleepcare app [50] found that 35 participants filled out >35 diaries, but few participants (n=4) completed <10% of the conversations. Kristjánsdóttir et al [51] found that 83.3% of participants completed 84 entries, yet 23.3% perceived the experience to be a burden.

User experience was measured using varying scales such as time of engagement and personal opinions of a particular app. The results varied. Although participants claimed that they found particular apps useful, this was not reflected by longitudinal engagement. There are multitudinous reasons why participants would deviate from the optimum engagement levels. For example, daily roles and responsibilities such as work, education, or parenting may not allow for optimum use. Other variables such as the lack of Wi-Fi connection and the loss of battery life may have hindered usage. These variables could possibly have skewed results. There were individuals who found diary entries a hindrance rather a help.

## Discussion

### Limitations

Although comprehensive, this review is not without limitations. This review is selective in its reporting as it only reviews studies employing an RCT design. This is not to refute the designs of excluded studies and their subsequent results, showing the efficacy of mHealth apps. In defense of this, the justification behind the selectiveness is stated within the rationale for review. Additionally, this review analyzed English language papers only; therefore, cross-cultural variations cannot be considered.

With regard to the topical therapy, CBT holds its own limitations. The efficacy of CBT is dependent on many variables. It requires a large amount of commitment, engagement, and cooperation from a patient. It can also be perceived time-consuming because of the regular sessions and frequent homework tasked.

### Future Research

Future research ought to work toward the long-term effectiveness of mHealth apps. Longitudinal studies ought to be carried out to assess usage against nonusage for both longer periods of time and greater follow-up periods. The necessity to eradicate technical problems before participant usage is key to fully assess the efficacy of mHealth apps without the interference of study formulated confounding variables.

There is a responsibility for both health professionals and media professionals alike to steer the general public away from the less scientifically created apps and toward regulated and sufficiently formulated and investigated apps. CBT therapists should be consulted and fully involved where apps are created using the principles of CBT to avoid information being misconstrued during usage. The comprehensive creation of mHealth apps with the relevant professionals will create a greater accessibility to effective treatment for the general public. Future apps would benefit from input from multidisciplinary teams during the design and development stages.

From a clinical perspective, medical information from professionals such as doctors, therapists, and counselors is imperative for inclusion in an app. This ensures that the app will distribute updated and medically validated advice and guidance. However, it would also be advantageous to include potential users of the app or those who have engaged with the therapy via varying mediums such as computerized, telephone, and face-to-face. This could provide valuable insights regarding the positive and negative aspects of all methods of deliverance, essentially guiding the development and improving the app. The inclusion of impartial charities and foundations that offer disorder-specific advice and guidance would benefit users. The field will of course take time to develop given the funding implications and time constraints involved with such research projects and the commercial value of other apps.

### Conclusions

This review specifically focused upon mHealth apps containing CBT content and RCT designs. In comparison to previous reviews, this review differs because of its focus upon one type of mHealth therapy app. Other reviews have explored mHealth apps in general, with reference to their purpose in health interventions [53]. This review investigated how one specific approach to therapy (CBT) has an impact when delivered as part of an mHealth app.

The results of this review highlight the effectiveness of mHealth apps that use CBT principles. Although the suitability of RCT studies assessing the efficacy of apps has been brought into question for reasons such as the inability to blind a participant to an mHealth intervention, the design has proven effective within this review. It is also worth noting that a patient’s motivation and commitment to therapy can determine the outcome of the therapy. Altering maladaptive cognition requires a willingness and devotion that individuals with mental health issues and vulnerability may not always possess. Although standard face-to-face CBT aims to be as personalized as possible, the same principles should be applied during the development of CBT mHealth apps.

Across a range of psychological issues, mHealth apps appear to repeatedly show improvements in symptom severity. Despite the clear effectiveness, the issue of longevity remains. With lower levels of effect at longer time intervals, questions may be raised over the long-term benefits of these mHealth apps. Similarly, the time constraints and funding issues for the creation of said apps is an issue with unregulated apps often taking precedence in the market place. Future research ought to address issues highlighted within this review and work toward more substantial datasets, long-term effects, and professionally created apps with the help of CBT therapists.

## Appendix

Multimedia Appendix 1 Searched Terms.

Multimedia Appendix 2 Cochrane's Risk of Bias.

Multimedia Appendix 3 Characteristics of mHealth apps using CBT.

## Abbreviations

BDI-II: Beck Depression Inventory-II
CBT: cognitive behavioral therapy
CBT-I: cognitive behavioral therapy for insomnia
iCBT: Internet-based CBT
mHealth: mobile Health
OCD: obsessive compulsive disorder
PHQ: Patient Health Questionnaire
PRISMA: preferred reporting items for systematic reviews and meta-analyses
PTSD: posttraumatic stress disorder
RCT: randomized controlled trial
SD: standard deviation
SG-CBT: self-guided cognitive behavioral therapy
TF-CBT: trauma-focused cognitive behavioral therapy
