# Supplementary material for: Assessing the Efficacy of Mobile Health Apps Using the Basic Principles of Cognitive Behavioral Therapy: Systematic Review
Source: J Med Internet Res. 2017 Nov 28;19(11):e399. doi: 10.2196/jmir.8598 (PMC5727354; doi:10.2196/jmir.8598)
Supplement: Multimedia Appendix 1 [file jmir_v19i11e399_app1.pdf]

Appendices

Appendix 1: Searched terms

Searched words	Conjunction	Disjunction	Examples of exact search terms
Cognitive, behaviour, behavioural, therapy, CBT, mobile phone(s), application, randomised, controlled, trial, RCT	and	N/A	Cognitive behavioural therapy AND mobile phone application randomised controlled trial<br>CBT AND mobile phone RCT<br>Mobile phone AND CBT RCT<br>Mobile phone application CBT RCT<br>CBT RCT AND mobile phones<br>CBT randomised trial AND mobile phones
