# Supplementary material for: Assessing the Efficacy of Mobile Health Apps Using the Basic Principles of Cognitive Behavioral Therapy: Systematic Review
Source: J Med Internet Res. 2017 Nov 28;19(11):e399. doi: 10.2196/jmir.8598 (PMC5727354; doi:10.2196/jmir.8598)
Supplement: Multimedia Appendix 2 [file jmir_v19i11e399_app2.pdf]

Appendix 2: Cochrane Risk of Bias Assessment of all Inclusive Studies

Trial	Random sequence generation	Allocation concealment	Incomplete outcome data	Selective reporting	Other biases	Description of other biases
Kuhn et al [45]	**Low risk**	**Low risk**	**Low risk**	**Low risk**	**High risk**	Monetary incentive
Ly, Asplund & Andersson [46]	**Low risk**	**Unclear** Allocation concealment was not noted within the study	**Low risk**	**Low risk**	**High risk**	First study of its kind
Ly et al [47]	**Unclear** Random sequence generation was not noted within the study	**Unclear** Allocation concealment was not noted within the study	**Low risk**	**Low risk**	**High risk**	Unequal gender split
Ly et al [48]	**Unclear** Random sequence generation was not noted within the study	**Unclear** Allocation concealment was not noted within the study	**Low risk**	**Low risk**	**Low risk**	N/A
Birney, Gunn, Russell & Ary [49]	**Unclear** Random sequence generation was not noted within the study	**Unclear** Allocation concealment was not noted within the study	**Low risk**	**Low risk**	**High risk**	Monetary incentive
Whittaker et al [50]	**Unclear**<br><br>Random sequence generation was not noted within the	**Low risk**	**High risk**<br><br>Only a fraction of the sample viewed all of the intended	**Low risk**	**Low risk**	N/A

	study		messages.<br><br>*“29.6% (n = 123) viewing most or all of the messages”*			
Horsch et al [51]	**Low risk**	**Low risk**	**High risk**<br><br>High attrition rates and there was no follow up for wait list	**Low risk**	**Low risk**	N/A
Kristjánsdóttir et al [52]	**Low risk**	**Unclear** Allocation concealment was not noted within the study	**Low risk**	**Low risk**	**Low risk**	N/A
