# Supplementary material for: Assessing the Efficacy of Mobile Health Apps Using the Basic Principles of Cognitive Behavioral Therapy: Systematic Review
Source: J Med Internet Res. 2017 Nov 28;19(11):e399. doi: 10.2196/jmir.8598 (PMC5727354; doi:10.2196/jmir.8598)
Supplement: Multimedia Appendix 3 [file jmir_v19i11e399_app3.pdf]

Multimedia Appendix 3:

Table 1.
Characteristics of mobile health (mHealth) apps using cognitive behavioral therapy (CBT).

Author (year) and app	Intervention and control group	Primary outcome measure	Main findings	Within-f and between-g effect size
Kuhn et al [45] PTSD[a] coach	Intervention n=62;Control n=58	PTSD symptoms	App users showed more improvement with depressive symptoms and coping	PTSD checklist civilian version *d*=0.14[b,e] *d*=0.86[b,d] *d*=1.09[c,d]
Ly et al [46] Unnamed	Intervention n=36; Control n=37	Stress	Mobile app stress intervention based on acceptance and commitment therapy reduces perceived stress and increases general health	12-item General Health Questionnaire: *d*=0.41[b,e] *d*=0.37[b,d] 14-item Perceived Stress Scale: *d*=0.50[b,e] *d*=0.62[b,d]
Ly et al [47] Unnamed	Intervention (a) Behavioral activation n=40; Intervention (b) Mindfulness n=41	Depression	The two interventions were equally effective; however, the behavioral activation intervention had	BDI-II[f]: *d*=0.24[b,e] *d*=0.03[c,e] *d*=1.83[b,d] *d*=1.19[c,d] PHQ[g]-9: *d*=0.28[b,e]

			more significant results for the more severely depressed	*d*=0.15[c,e]<br>*d*=1.63[b,d]<br>*d*=1.14[c,d]
Ly et al [48]<br>Unnamed	Intervention (a)<br>Blended treatment<br>(app + 4 FTF[h] therapy sessions)<br>n=46;<br>Intervention (b)<br>Full behavioral activation<br>(no app + 10 FTF therapy sessions)<br>n=41	Depression	Inconclusive findings	BDI-II:<br>*d*=-0.13[b,e]<br>*d*=-0.10[c,e]<br><br>Intervention (a)<br>*d*=1.40[b,d]<br>*d*=1.35[c,d]<br><br>Intervention (b)<br>*d*=1.47[b,d]<br>*d*=1.44[c,d]
Birney et al [49]<br>MoodHacker	Intervention (a)<br>Mobile app<br>n=150;<br>Intervention (b)<br>Alternate care<br>n=150	Depression	The app produced significant effects on depressive symptoms	PHQ:<br>*d*=0.14[b,e]<br><br>Intervention (a):<br>*d*=0.93[b,d]<br><br>Intervention (b):<br>*d*=0.92[b,d]
Whittaker et al [50]<br>MEMO	Intervention n=426; Control n=429	Depression	Significantly assisted participants to rid their selves of negative thoughts	Increased positivity:<br>*d*=1.19[b,e]

Horsch et al [51]<br>The Sleepcare app	Intervention<br>Cognitive behavioral therapy for insomnia app<br>n=74<br><br>Wait-list Control<br>n=77	Insomnia severity	Significant improvement in relatively mild insomnia	Insomnia severity inventory:<br>*d*=-0.66[b,e]<br>*d*=1.33[b,d]
Kristjánsdóttir et al [52]<br>Unnamed	Intervention n=62; Control n=65	Chronic pain acceptance	No between group effect but there was a within group effect on the intervention. Presents slight findings suggesting improvement in chronic pain acceptance	Pain Catastrophizing Scale:<br>*d*=0.61[b,d]<br>*d*=1.02[c,d]<br>*d*=0.03[b,e]<br>*d*=0.35[c,e]

[a]PTSD: posttraumatic stress disorder.

[b]post test.

[c]follow-up.

[d]within-group effect.

[e]between-group effect.

[f]BDI-II: Beck Depression Inventory-II.

[g]PHQ: Patient Health Questionnaire.

[h]FTF: face-to-face.

References

45. Kuhn, E., Kanuri, N., Hoffman, J. E., Garvert, D. W., Ruzek, J. I., & Taylor, C. B. A randomized controlled trial of a smartphone app for posttraumatic stress disorder symptoms. J Consult Clin Psychol. 2017 Mar;85(3):267-273. doi: 10.1037/ccp0000163. PMID: 28221061
46. Ly, K. H., Asplund, K., & Andersson, G. (2014). Stress management for middle managers via an acceptance and commitment-based smartphone application: A randomized controlled trial. Internet Interventions, 1(3), 95-101.
47. Ly, K. H., Trüschel, A., Jarl, L., Magnusson, S., Windahl, T., Johansson, R., ... & Andersson, G. Behavioural activation versus mindfulness-based guided self-help treatment administered through a smartphone application: a randomised controlled trial. *BMJ Open. 2014 Jan 9;4(1):e003440. doi: 10.1136/bmjopen-2013-003440. PMID: 24413342*
48. Ly, K. H., Topooco, N., Cederlund, H., Wallin, A., Bergström, J., Molander, O., ... & Andersson, G. Smartphone-supported versus full behavioural activation for depression: a randomised controlled trial. PLoS One. 2015 May 26;10(5):e0126559. doi: 10.1371/journal.pone.0126559. eCollection 2015. PMID: 26010890
49. Birney, A. J., Gunn, R., Russell, J. K., & Ary, D. V. MoodHacker mobile Web app with email for adults to self-manage mild-to-moderate depression: randomized controlled trial. JMIR Mhealth Uhealth. 2016 Jan 26;4(1):e8. doi: 10.2196/mhealth.4231. PMID: 26813737
50. Whittaker, R., Merry, S., Stasiak, K., McDowell, H., Doherty, I., Shepherd, M., ... & Rodgers, A. MEMO—a mobile phone depression prevention intervention for adolescents: development process and postprogram findings on acceptability from a randomized controlled trial. J Med Internet Res. 2012 Jan 24;14(1):e13. doi: 10.2196/jmir.1857. PMID: 22278284
51. Horsch, C. H., Lancee, J., Griffioen-Both, F., Spruit, S., Fitrianie, S., Neerincx, M. A., ... & Brinkman, W. P. Mobile phone-delivered cognitive behavioral therapy for insomnia: a randomized waitlist controlled trial. *J Med Internet Res. 2017 Apr 11;19(4):e70. doi: 10.2196/jmir.6524. PMID: 28400355*
52. Kristjánsdóttir, Ó. B., Fors, E. A., Eide, E., Finset, A., Stensrud, T. L., van Dulmen, S., ... & Eide, H. A smartphone-based intervention with diaries and therapist-feedback to reduce catastrophizing and increase functioning in women with chronic widespread pain: randomized controlled trial. *J Med Internet Res. 2013 Jan 7;15(1):e5. doi: 10.2196/jmir.2249.* PMID: 23291270
